# Comparing the performance of urine and copro-antigen detection in evaluating *Opisthorchis viverrini* infection in communities with different transmission levels in Northeast Thailand

**DOI:** 10.1371/journal.pntd.0007186

**Published:** 2019-02-08

**Authors:** Chanika Worasith, Chompunoot Wangboon, Kunyarat Duenngai, Nadda Kiatsopit, Kulthida Kopolrat, Anchalee Techasen, Jiraporn Sithithaworn, Narong Khuntikeo, Watcharin Loilome, Nisana Namwat, Puangrat Yongvanit, Elizabeth J. Carlton, Paiboon Sithithaworn

**Affiliations:** 1 Department of Parasitology, Faculty of Medicine, Khon Kaen University, Khon Kaen, Thailand; 2 Cholangiocarcinoma Research Institute, Khon Kaen University, Khon Kaen, Thailand; 3 Biomedical Science Program, Graduate School, Khon Kaen University, Khon Kaen, Thailand; 4 Department of Public Health, Faculty of Science and Technology, Phetchabun Rajabhat University, Phetchabun, Thailand; 5 Faculty of Associated Medical Sciences, Khon Kaen University, Khon Kaen, Thailand; 6 Faculty of Medicine, Mahasarakham University, Mahasarakham, Thailand; 7 Department of Surgery, Faculty of Medicine, Khon Kaen University, Khon Kaen, Thailand; 8 Department of Biochemistry, Faculty of Medicine, Khon Kaen University, Khon Kaen, Thailand; 9 Department of Environmental and Occupational Health, Colorado School of Public Health, University of Colorado, Anschutz, Aurora, Colorado, United States of America; Seoul National University College of Medicine, REPUBLIC OF KOREA

## Abstract

To combat and eventually eliminate the transmission of the liver fluke *Opisthorchis viverrini*, an accurate and practical diagnostic test is required. A recently established urine antigen detection test using monoclonal antibody-based enzyme-linked-immunosorbent assay (mAb-ELISA) has shown promise due to its high diagnostic accuracy and the use of urine in place of fecal samples. To further test the utility of this urine assay, we performed a cross sectional study of 1,043 people in 3 opisthorchiasis endemic communities in northeast Thailand by applying urine antigen detection together with copro-antigen detection methods. The quantitative formalin-ethyl acetate concentration technique (FECT) was concurrently performed as a reference method. The prevalence of *O*. *viverrini* determined by urine antigen detection correlated well with that by copro-antigen detection and both methods showed 10–15% higher prevalence than FECT. Within the fecal negative cases by FECT, 29% and 43% were positive by urine and copro-antigen detection, respectively. The prevalence and intensity profiles determined by antigen detection and FECT showed similar patterns of increasing trends of infection with age. The concentration of antigen measured in urine showed a positive relationship with the concentration of copro-antigen, both of which were positively correlated with fecal egg counts. The data observed in this study indicate that urine antigen detection had high diagnostic accuracy and was in concordance with copro-antigen detection. Due to the ease and noninvasiveness of sample collection, the urine assay has high potential for clinical diagnosis as well as population screening in the program for the control and elimination of opisthorchiasis.

## Author summary

Opisthorchiasis, caused by an infection with the liver fluke, *Opisthorchis viverrini*, is a neglected tropical disease endemic in Southeast Asia, particularly Thailand and Lao PDR. *O*. *viverrini* as well as *Clonorchis sinensis* have been classified as group I biological carcinogenic agents for cholangiocarcinoma (CCA). Due of the impact of control programs, the prevalence and worm burden in endemic communities have been reduced and this has caused the conventional fecal examination to be less sensitive and unreliable. In order to improve the diagnosis and to move towards the elimination of liver fluke to reduce CCA, we evaluated a novel urine antigen detection method by mAb-enzyme-linked immunosorbent assay for the diagnosis and screening of opisthorchiasis in endemic communities in northeast Thailand. We concurrently applied two coprological methods for antigen detection and fecal examination by formalin–ethyl acetate concentration technique, a reference method for comparison. Urine and copro-antigen detection had comparable diagnostic accuracy and both methods performed better than the fecal examination. Because of the ease and acceptance of urine specimen collection and handling, urine antigen detection has a high potential for the diagnosis and mass screening of opisthorchiasis in control and elimination programs.

## Introduction

Opisthorchiasis is a neglected tropical disease caused by an infection with a small human liver fluke *Opisthorchis viverrini*. Based on epidemiological and experimental evidence, *O*. *viverrini* as well as a closely related species, *Clonorchis sinensis*, were classified as group I biological carcinogenic agents [1]. Opisthorchiasis is known to cause important public health problems in mainland Southeast Asia, particularly in Thailand, Lao PDR, Cambodia and Vietnam [2, 3]. The current transmission landscape consists of a light and uneven distribution of *O*. *viverrini* infection in endemic areas, a feature that has consequences for diagnostic accuracy, as well as the role of *O*. *viverrini* as risk factor of cholangiocarcinoma (CCA) [4, 5]. For the success of any liver fluke control and elimination program, particularly for mapping and facilitating drug treatment, an improved diagnostic method that is suitable to the current endemic conditions is needed [6, 7].

To date, definitive diagnosis of *O*. *viverrini* infection is achieved by finding parasite eggs in feces, however, such parasitological diagnosis has many drawbacks including false positivity caused by confusion with the eggs of minute intestinal flukes, or by false negativity in light infections and in biliary duct obstruction where no eggs can be detected in feces. Repeated stool examination is required to increase the reliability of the results [8–10], but the cost and requirement of an expert microscopist make this method less practical.

Previously molecular and immunological-based diagnostic methods have been developed and applied for the diagnosis of opisthorchiasis [11–15]. Although these methods have provided a better diagnostic performance compared with the parasitological method, they have several drawbacks regarding their sensitivity and specificity according to the abundance of the target genes, antigens or antibodies, and also the presence of inhibitors in clinical samples [7, 16]. An antibody-based approach for the detection of circulating antibody has limitations due to the cross reactive nature of the antigens used [17–21] and a positive result does not always indicate active infection by the parasite [19, 22, 23].

Unlike antibody detection, an antigen detection assay detects a current and viable parasite infection which better reflects the infection status in opisthorchiasis patients. In this regard, monoclonal antibody-based enzyme linked immunosorbent assays (mAb-ELISA) for detecting parasite antigen in fecal samples (copro-antigen) have been introduced and verified in clinical samples [24, 25]. The mAb-ELISA provides several advantages over conventional methods since it has a higher specificity, good reproducibility, and can be prepared in large quantities [26]. In addition to previous studies [24, 25], our group has reported an improved protocol for copro-antigen detection with high diagnostic performance [27]. Subsequently, in 2015, we reported a novel antigen detection method using urine samples for the diagnosis of opisthorchiasis [28]. Both urine and copro-antigen detection yielded a high diagnostic performance compared with standard fecal examination, but a comparison between these antigen detection methods in the same sample population has not been reported.

In this study, we aim to assess the field application of this method, comparing the diagnostic performances of urine antigen detection with that of copro-antigen detection. The formalin-ethyl acetate concentration technique (FECT) was used as a reference method. This study was conducted with the residents of 3 opisthorchiasis endemic communities in Northeast Thailand that showed varying prevalence and intensity of *O*. *viverrini* infection. We also investigate the quantitative relationships between the levels of *O*. *viverrini* antigens in urine and fecal samples and the fecal egg counts determined by the formalin-ethyl acetate concentration technique.

## Materials and methods

### Ethical statement

The human subject protocol used in this study was approved by the Human Ethics Committee of Khon Kaen University, Thailand (reference number HE561478). Written informed consent was obtained from individual subjects and those with the *O*. *viverrini*-positive examination by FECT or antigen detection methods were treated with a single oral dose of praziquantel (40 mg/kg body weight).

The experimental protocols for laboratory animal handling for monoclonal and polyclonal antibody production and also parasite antigen production were approved by the Institutional Animal Ethical Committee, Khon Kaen University (AEKKU-NELAC 26/2558). All animals were anesthetized with isoflurane inhalation before immunization. For euthanization, the animals were anesthetized by isoflurane and sacrificed by drawing of blood from the heart. No animals were demonstrated the severe health problems during in this study. The procedure was performed in strict accordance with the guidelines for the Care and Use of Laboratory Animals of the National Research Council of Thailand.

### Study area and study participants

This prospective cross-sectional study began in March 2015 and ended in July 2016. The eligibility criteria of the participants were; (i) participants who were native residents in Ban Wa sub-district in Khon Kaen Province (KK), Tao Ngoi sub-district, Sakon Nakhon province (SK) and Nong Khon Thai sub-district, Chaiyaphum province (CP), Northeast Thailand; (ii) participants who agreed to provide both feces and urine on the same day for index and standard reference tests; (iii) participants who were apparently healthy and had no clinical signs or symptoms. The sample size of this study was calculated based on the average proportion of positive *O*. *viverrini* cases (28%) with the derivative set at 1.96 and corresponding to a 95% confident level ± 5%. The calculated sample size was 930 participants with a potential loss of sample submission of 20% so that the number of participants in three localities to be recruited was 1,116.

**Fig 1:**
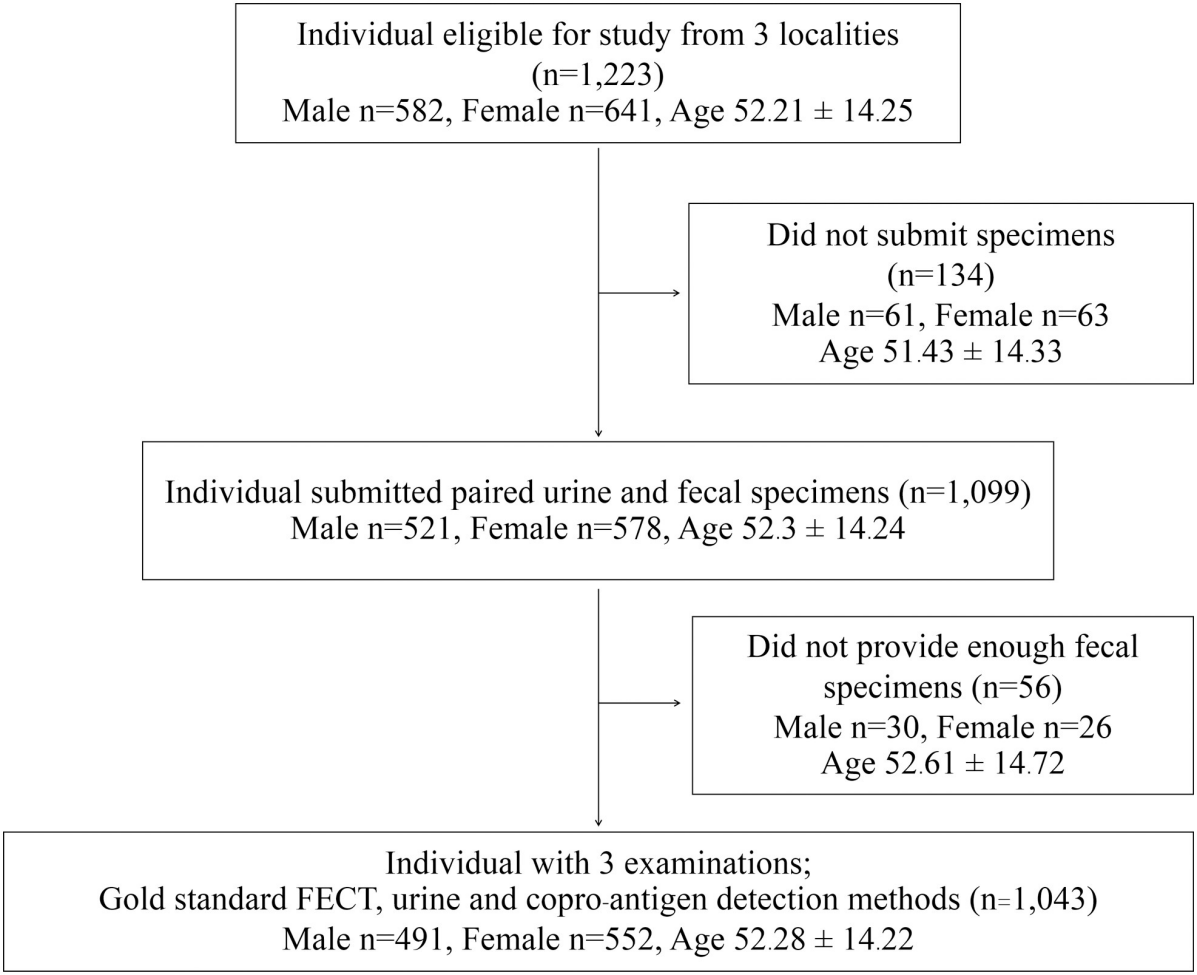
Flow diagram of study participants and sample collection. The flowchart shows the number of participants included and excluded from the project comparing diagnostic methods for human opisthorchiasis by formalin ethyl-acetate concentration technique (FECT) and antigen detections in urine and fecal sample.

Of the 1,233 participants originally enrolling, 180 were excluded because they either failed to submit clinical specimens (n = 134) or submitted inadequate specimens (n = 56). A total of 1,043 participants fulfilled the inclusion criterion and provided complete clinical samples (Fig 1). There was no statistical difference in demographics data (age, sex, etc.) between the recruited and the excluded participants.

### Clinical samples

Feces (10 g) and samples of the first morning, mid-stream urine (5 ml) were collected from the project participants in standard plastic containers and kept in a chilled box (4°C-8°C) during transportation to the laboratory within one day of collection. At the laboratory, each feces sample was separated into 2 parts: (i) aliquoted and processed for parasite examination by the formalin ethyl-acetate concentration technique (FECT), (ii) kept in 1.5 ml tube and stored at -20°C for copro-antigen detection. The urine samples were centrifuged at 1,500 rpm at 4°C for 15 minutes and the cleared supernatants were aliquoted into new vials and stored at -20°C until required for mAb-ELISA.

### Faecal examination by the quantitative formalin-ethyl acetate concentration technique (FECT)

The procedure for quantitative FECT was similar to the procedure detailed in the previous report [29]. In brief, 2 grams of fresh stool were fixed in 10% formalin and kept at room temperature until processing. After vigorous shaking, the specimens were centrifuged at 2,500 rpm for 5 minutes and the sediment re-suspended with NSS and strained through two layers of gauze. The tube was then centrifuged at 2,500 rpm for 5 minutes and the supernatant discarded. Seven milliliters of 0.85% saline were added to the sediment and mixed, then 3 ml of ethyl acetate were added to the sample tube, mixed thoroughly, and centrifuged at 2,500 rpm for 5 minutes. After removal of the top three layers, the sediment was re-suspended with 1 ml of 10% formalin. The final suspension was examined blind by two parasitologists and the number of *O*. *viverrini* eggs were counted in 2 drops sampled from the total suspension. The number of eggs per gram of feces (EPG) was calculated to determine the intensity of *O*. *viverrini* and other parasitic infection.

### Antigen detection by monoclonal antibody-based enzyme linked immunosorbent assay (mAb-ELISA)

#### Sample preparation and pretreatment

The frozen urine and feces samples were thawed at room temperature prior to processing. For fecal sample, phosphate buffered saline (PBS) pH 7.2 was added (1: 3 by volume), mixed thoroughly by vortexing and centrifuged at 2,000 rpm at 4°C for 30 minutes. The resulting supernatants were transferred into new vials and centrifuged at 10,000 rpm at 4°C for 5 minutes. The urine samples and also the final supernatants of fecal samples were pretreated with an equal volume of 4% trichloroacetic acid (TCA) to remove interfering components as described previously [27, 28, 30]. The sample tubes were mixed, vortexed and then incubated for 20 minutes at room temperature. Finally, the mixture was neutralized with an equal volume of 0.244 M carbonate buffer (pH 9.6).

### Procedure for antigen detection by mAb-ELISA

The protocols for mAb-ELISA for *O*. *viverrini* antigen measurements in urine and fecal samples were similar to those previously described [27, 28]. Polystyrene microtiter plates (NUNC, Roskilde, Denmark) were sensitized overnight at 4°C with 100 μl/well of 5 μg/ml of mAb diluted in 50 mM bicarbonate buffer pH 9.6. After coating, the plates were washed three times with normal saline containing 0.05% Tween 20 (NSST) and uncoated sites were blocked with a solution of 5% dried skimmed milk in 50 mM bicarbonate buffer pH 9.6. After incubation for 1 hour at 37°C, TCA-pretreated urine and fecal extracted samples were added and the plates (100 μl/well) were incubated at 37°C for 1 hour. Next, the plates were washed five times with NSST and purified IgG rabbit against crude *O*. *viverrini* antigen (10 μg/ml) and 2% dried skimmed milk in PBST was added and incubated at 37°C for 1 hour. After that, 100 μl/well of biotinylated goat anti-rabbit IgG conjugate (1: 4,000) in 2% dried skimmed milk in PBST were added (Invitrogen, CA, USA) and the plates were incubated at 37°C for 1 hour. After washing three times, horseradish peroxidase-conjugated streptavidin (GE Healthcare, Buckinghamshire, UK) (dilution 1: 5,000) in PBST was added and the plates were incubated at 37°C for 1 hour. Plates were washed and the substrate (*o*-phenylenediamine hydrochloride) solution (Sigma, St. Louis, MO, USA) was added and incubated for 20 minutes in the dark at room temperature. The enzyme reaction was stopped with 100 μl/well of 2M sulfuric acid (H_2_SO_4_) and the optical densities (OD) were read spectrophotometrically at 492 nm with an ELISA reader (Tecan Sunrise Absorbance Reader, Austria). Two well-trained laboratory staff were responsible for the simultaneous sample analysis of the index tests. During the laboratory procedure, the sample IDs were blinded and the laboratory staff had no knowledge of the sample subjects.

### Receiver operation characteristic curve (ROC curve)

Known negative and positive urine samples and also fecal samples for *O*. *viverrini* infection determined by FECT were used to construct a Receiver Operation Curve (ROC). The cutoff points for diagnosis by mAb-ELISA were calculated using MedCalc software version 9.6.3 (MedCalc Software, Ostend, Belgium).

The OD values were transformed to antigen concentrations based on the standard curves for urine and feces using spiked *O*. *viverrini*-ES antigen extract with varying concentrations starting with 5,000 ng and followed by two-fold serially dilutions to produce a standard calibration curve. The separated cut off value was 19.4 ng/ml for urine and 61.2 ng/ml copro antigen detection methods. These were used to determine negative and positive tests. The sensitivity, specificity, and positive and negative predictive values with 95% confidence intervals were calculated and compared between each sample using standard parasitological methods as a reference. In addition, a composite reference standard method which combined both parasitological, urine or fecal antigen detection methods as a reference method was used to assess the diagnostic performance of each method.

### Cross reactivity

In order to assess the specificity of the mAb-ELISA for *O*. *viverrini* antigen detection in both urinary and copro-antigen detection method, cross reactivity with positive samples of other parasitic infections determined by FECT was assessed.

For urinary antigen detection, the other parasite infections, i.e. *Strongyloides stercoralis* (n = 40), minute intestinal fluke (n = 17), hookworms (n = 10), *Taenia* sp. (n = 5), *Echinostoma* sp. (n = 8) and *Trichuris trichiura* (n = 5), were tested for cross reactivity. Cross reactivity of copro-antigen detection was performed with *Strongyloides stercoralis* (n = 40), minute intestinal fluke (n = 17), hookworms (n = 10) and *Taenia* sp. (n = 5).

### Statistical analysis

Data recorded in the case report forms were entered into a computer using the Microsoft Excel program and analyzed using SPSS v.22 (International Business Machines, USA). Helminth species-specific fecal egg counts were transformed into number of eggs per gram of feces (EPG). Kendall’s tau-b correlation test was used to determine the correlation between urinary-antigen and copro-antigen concentration and also EPG. Performance of the test in terms of sensitivity, specificity, and predictive values was calculated as described elsewhere [31]. The OD values were transformed to a ratio between the OD of the samples and the OD of reference urine or fecal extracted samples. The reliability of urine and copro-antigen detection methods by mAb-ELISA for the diagnosis of opisthorchiasis was analyzed using odds ratios (OR) with 95% confidence intervals (CI) using logistic regression. A statistically significant level was set as p<0.05. We used the following guidelines to interpret the kappa values; ≤ 0 indicating no agreement, 0–0.2, poor agreement; 0.21–0.4; fair agreement; 0.41–06, moderate agreement; 0.61–0.8, good agreement; and 0.81–1.0, excellent agreement [32].

## Results

### Prevalence and intensity of *O*. *viverrini* infection

As shown in Table 1, based on FECT, the prevalence of *O*. *viverrini* infection in the three localities was 29–77% with an overall prevalence of 41%. The highest prevalence was in Nong Khon Thai sub district, Chiyaphum (CP), followed by Tao Ngoi subdistrict, Sakon Nakhon (SK) and the lowest was in Ban Wa sub-district, Khon Kaen (KK). The intensity of infection measured by fecal egg counts (EPG) were similar in the three localities which had mainly either no or light infection (EPG<50) with approximately 13% having an EPG>100. The average intensity of infection by locality was 25–127 EPG and the overall intensity was 54 EPG. Other parasitic infections occurred in <9% of the samples and the prevalence by order were *S*. *stercoralis*, MIFs, hookworm and *Taenia* spp. By urine antigen detection, the prevalence of *O*. *viverrini* was 46–77% by locality and the overall prevalence was 54%. The antigen concentration was 27–40 ng/ml of urine with an average of 31 ng/ml in all three localities. In the case of copro-antigen detection, the prevalence of *O*. *viverrini* was 48–78% with an overall value of 56%. The antigen concentration in feces was 75–90 ng/ml of feces and an average concentration of 81 ng/ml.

**Table 1:** Characteristics of study participants and the prevalence and intensity of *O*. *viverrini* determined by quantitative FECT, urine and copro-antigen detection in the study localities in northeast, Thailand.

Variable	KK	SK	CP	All sites
Participants	558	281	204	1,043
Number of males (%)	287 (51)	97 (35)	107 (52)	491 (47)
Number of females (%)	271 (49)	184 (65)	97 (48)	552 (53)
Age (Mean ± SD)	53 ± 12.7	48.5 ± 16.3	55.0 ± 13.6	52.2 ± 14.1
Reference standard (FECT)				
Prevalence no+ve (%)	160 (29)	105 (37)	158 (77)	423 (41)
Infection intensity (EPG)				
0	367 (66)	154 (55)	27 (14)	548 (53)
1–50	101 (18)	48 (17)	62 (30)	211 (20)
51–100	22 (4)	11 (4)	39 (19)	72 (7)
>100	37 (7)	46 (16)	57 (28)	140 (13)
Mean EPG (Geometric Mean ± SE)	25.3 ± 4.8	59.7 ± 10.1	127.1 ± 15.9	54.5 ± 5.0
*Other parasites infection	31 (6)	22 (8)	19 (9)	72 (7)
Urinary antigen test				
Prevalence no+ve (%)	257 (46)	152 (54)	157 (77)	566 (54)
Antigen concentration (Geometric Mean ± SE)	27.2 ± 1.2	32.9 ± 1.7	40.9 ± 4.6	31.4 ± 1.2
Copro-antigen test				
Prevalence no+ve (%)	266 (48)	156 (56)	160 (78)	582 (56)
Antigen concentration (Geometric Mean ± SE)	75.7 ± 2.8	85.1 ± 5.2	90.2 ± 4.2	81.1 ± 2.2

KK: Ban Wa sub-district, Khon Kaen Province

SK: Tao Ngoi sub-district, Sakon Nakhon Province

CP: Nong Khon Thai sub-district, Chaiyaphum Province

*Other parasites included *Strongyloides stercoralis*, minute intestinal flukes (MIFs), hookworms and *Taenia* spp.

### Age-prevalence and intensity profiles

**Fig 2:**
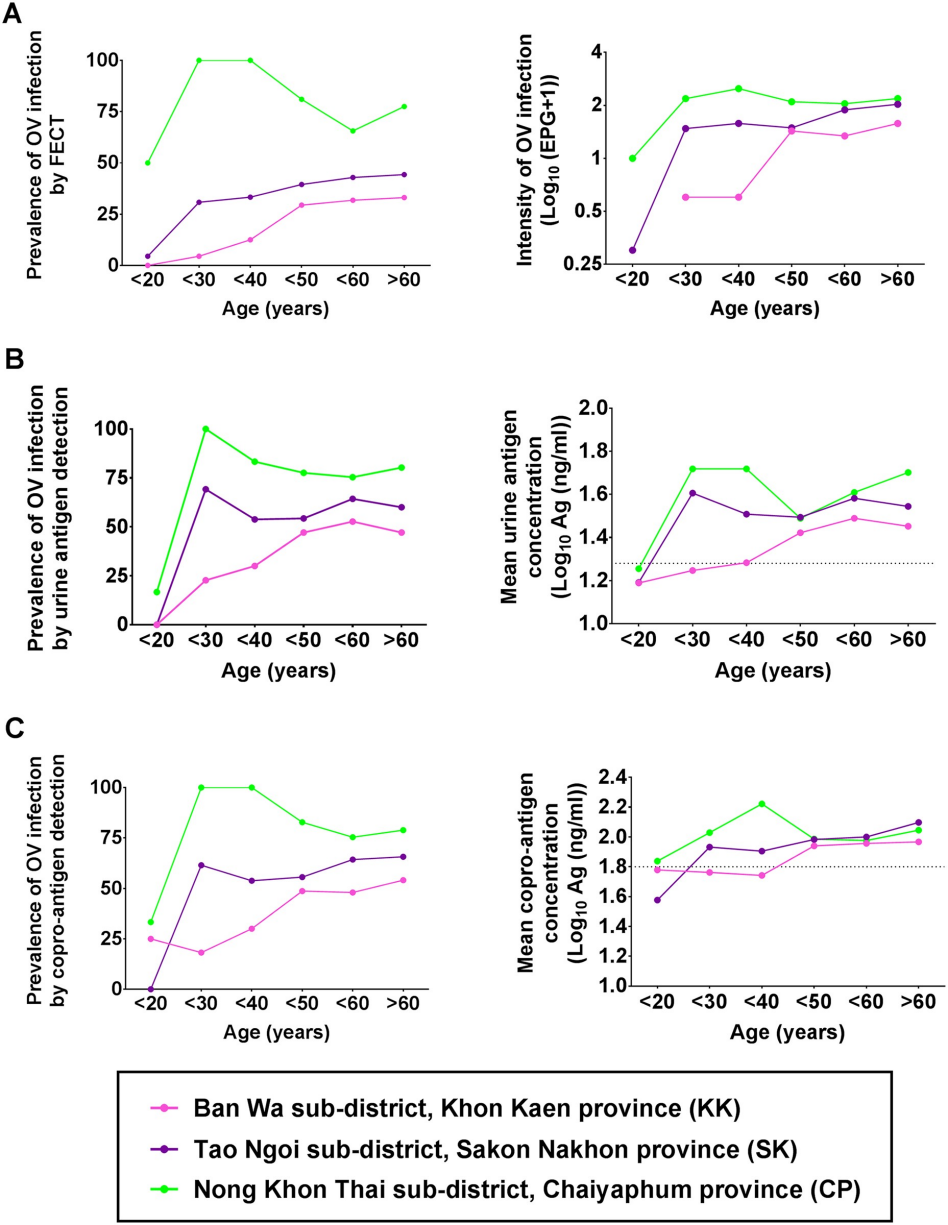
Age prevalence and intensity profiles of *O*. *viverrini* infection determined by different diagnostic methods in 3 different localities. (A) Determination of infection by FECT. (B) Urine antigen detection and (C) copro antigen detection measured by mAb-ELISA. The prevalence profiles of opisthorchiasis (left panel) and intensity of infection (right panel) are illustrated according to the age group and locality.

As shown in Fig 2, the *O*. *viverrini* infection determined by FECT showed the prevalence profile that peaked at 30–40 years in CP and SK, but in KK the prevalence increased slowly and peaked at an age >60 years. The intensity profiles increased steeply from 20–30 years and stabilized thereafter in CP and SK. In KK the intensity increased slowly with age and reached a plateau at age 50 years onwards.

The age-prevalence profiles of *O*. *viverrini* by urine and copro-antigen detection (Fig 2B and 2C) shared a similar pattern in CP and SK where they peaked at age <30 years and plateaued thereafter. In the case of KK, the prevalence increased steadily with age and became a maximum at age <50 years. The age-intensity profiles measured by urine and copro-antigen were similar to those by FECT, being highest at age <30 years and slightly less at age <50 years in CP and SK. The intensity profile for KK increased slowly with age and was highest at age <60 years.

### Quantitative relationship between urinary and copro-antigen concentrations of spiked samples

In order to construct the standard curves for the calculation of antigen concentrations in urine and fecal samples, measurement of antigens in spiked excretory-secretory antigen of *O*. *viverrini* in urine and feces was performed using mAb-ELISA. The relationships between the concentrations of in urine and fecal samples and OD values obtained from mAb-ELISA were assessed by linear regression models. The best-fit linear regression equations for urinary and copro-antigen detection was y = 0.856x-0.831 and y = 0.653x-0.725, respectively. These equations were used to calculate the concentration of antigen in the clinical samples from the project participants (S1 Fig).

### Levels of *O*. *viverrini* antigen in urine and feces in relation to intensity of infection

In order to determine the effect of EPG on the level of antigen detected in urine and feces, the participants from all localities were combined and then separated into 4 different intensity groups based on EPG determined by FECT (Table 2). Based on the urine and copro-antigen assays, the positive infection rates were 31% of egg-negative subjects by FECT. In egg-positive groups, the positive infection rates were 83–97% for urine and 83–98% for copro-antigen detections. Overall, urine and copro-antigen detections yielded similar positive rates and both assays yielded 14–15% higher positive rates than FECT.

**Table 2:** Positive rates of *O*. *viverrini* determined by urinary and copro-antigen detection in different groups of participants stratified by intensity of infection (EPG).

Intensity of O. viverrini infection (EPG)	N	*O*. *viverri* positive by	
		Urine antigen n (%)	Copro-antigen n (%)
0	620	191 (31)	195 (31)
1–50	211	176 (83)	189 (90)
51–100	72	63 (88)	60 (83)
>100	140	136 (97)	138 (98)
**Total**	**1,043**	**566 (55)**	**582 (56)**

**Fig 3:**
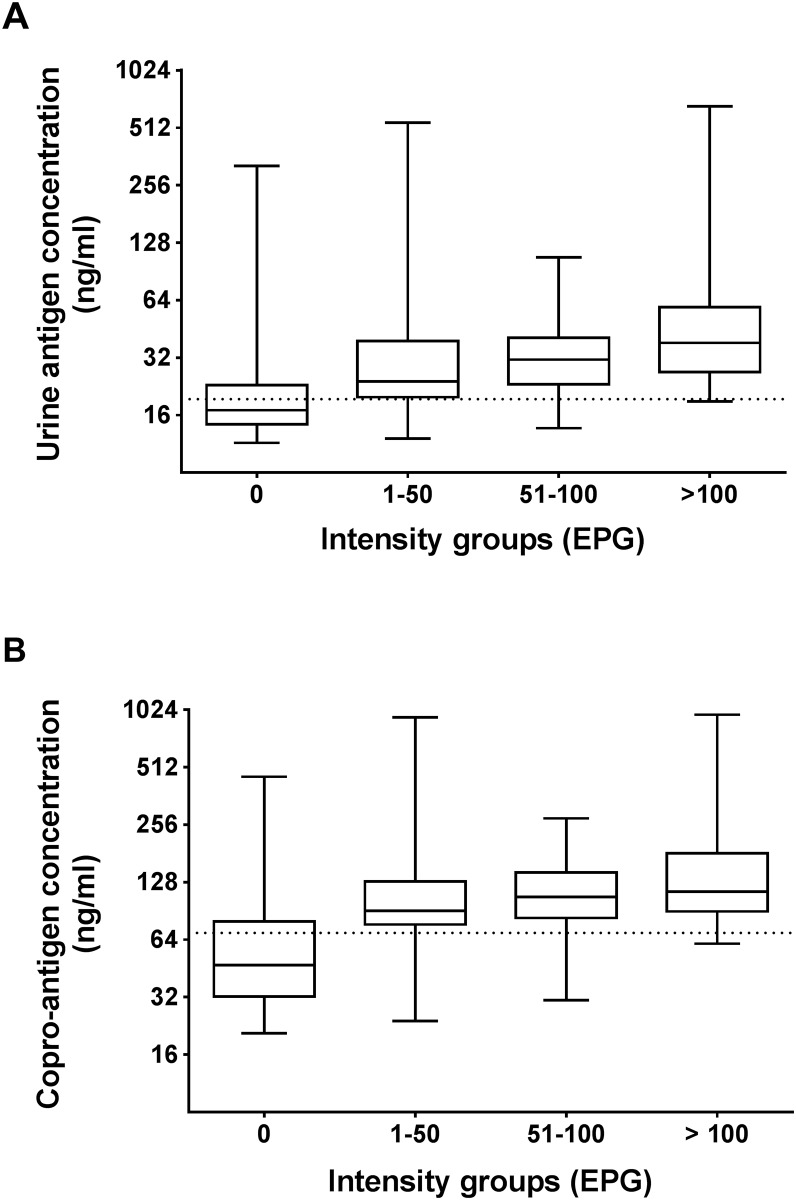
Relation between *O*. *viverrini* antigen levels and intensity of *O*. *viverrini* (EPG). (A) The profiles of urine antigen levels (ng/ml) in different intensity groups of *O*. *viverrini* (EPG) (Kendall’s tau-b correlation; P <0.001); (B) The profiles of copro-antigen levels (ng/ml) in different intensity groups of *O*. *viverrini* (EPG) (Kendall’s tau-b correlation; P <0.001).

The concentrations of urinary and copro-antigen showed a significant positive correlation with increasing intensity of *O*. *viverrini* (EPG) (Kendall’s tau-b, p < 0.001; Fig 3A and 3B).

### The relationship between urine and copro-antigen concentrations and their diagnostic performances

**Fig 4:**
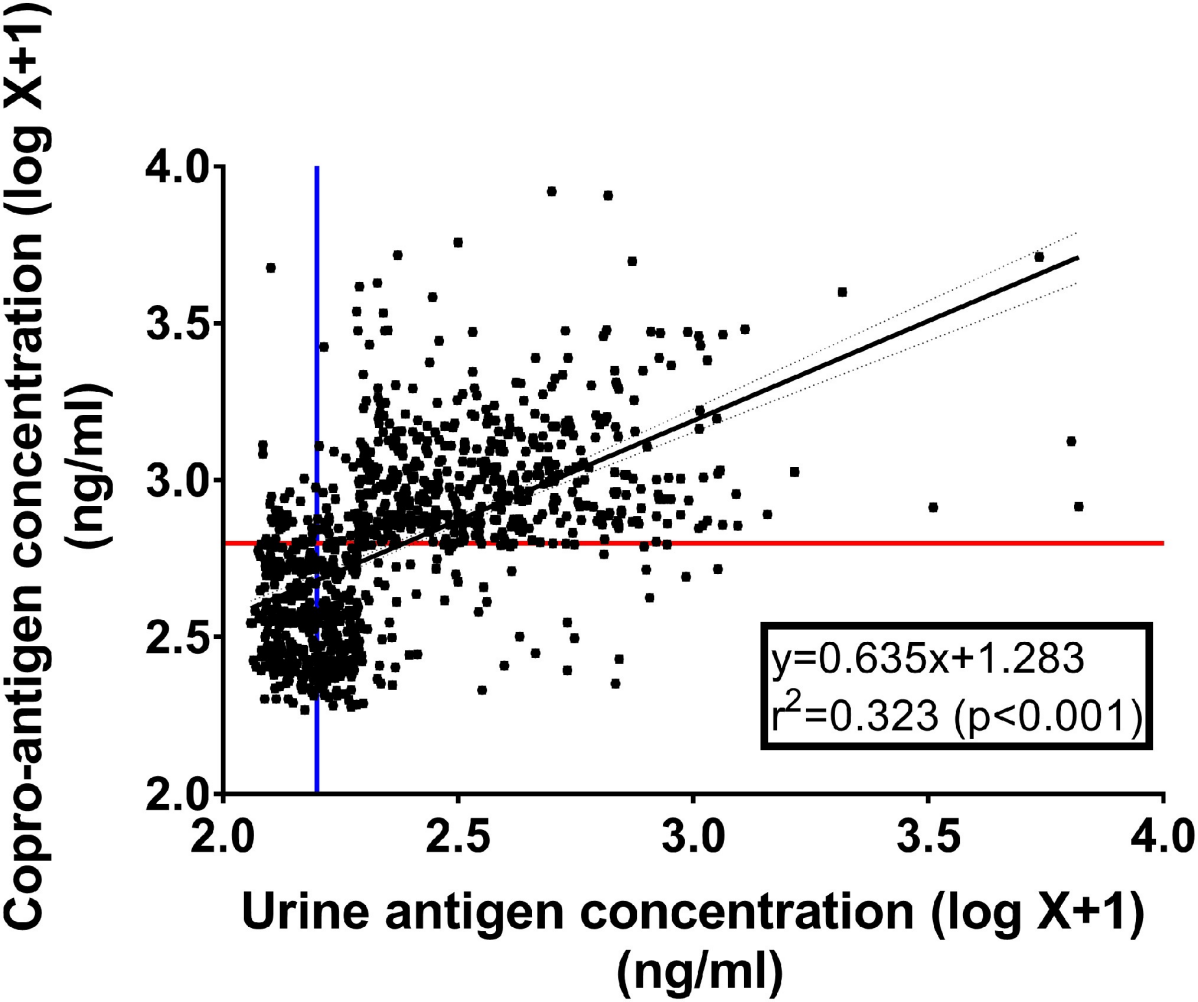
Relationship between *O*. *viverrini* antigen concentrations in urine and feces determined by mAb-ELISA. (Kendall’s tau-b correlation; p <0.001). Data shown are observed values (rectangles) and the linear regression line between urine and copro-antigen concentrations. The vertical (blue) and horizontal (red) lines are cut off values for urine and copro-antigen, respectively.

Based on 1,043 participants who provided both fecal and urine samples for analysis of *O*. *viverrini* antigen concentrations, there was a significant positive correlation between urine and copro-antigen concentrations (Fig 4, R^2^ = 0.323, p < 0.001). The best fit linear regression equation was y = 0.635+1.283 where y = copro-antigen concentration (log-transformed value; ng/mL and x = urine antigen concentration (log-transformed value; ng/mL).

Table 3 shows the diagnostic performance of urine and copro-antigen detections determined in field-collected samples by using FECT as a gold standard. By locality, urine antigen detection exhibited a sensitivity between 86–90% and a specificity between 56–71%. The overall sensitivity in 3 localities was 89.5% (95% CI = 86.1–92.3) and specificity 71.2% (95% CI = 67.6–74.7). The diagnostic performance of copro-antigen detection was comparable to that for urine assay with the overall 90.7% sensitivity and 70% specificity.

**Table 3:** Diagnostic performance of antigen detection by urine and copro-antigen detection in comparison with the gold standard FECT (n = 1043).

Tested method	Locality	Sensitivity (95% CI)	Specificity (95% CI)	°PPV	°NPV
Urine antigen detection	KK	89.4 (83.5–93.7)	71.4 (66.6–75.8)	55.6	94.4
	SK	90.5 (83.2–95.3)	67.6 (60.2–74.5)	62.5	92.2
	CP	86.1 (79.7–91.1)	56.5 (41.1–71.1)	82.7	54.2
	Overall	89.5 (86.1–92.3)	71.2 (67.6–74.7)	66.2	89.7
Copro-antigen detection	KK	94.4 (89.6–97.4)	71.6 (66.9–76.0)	57.2	96.9
	SK	92.4 (85.5–96.7)	66.5 (59.0–73.4)	62.2	93.6
	CP	88.0 (81.9–92.6)	54.3 (39.0–69.1)	86.9	56.8
	Overall	90.7 (87.5–93.4)	70.0 (66.3–73.5)	66.7	92.2

°Positive Predictive Value (PPV), Negative Predictive Value (NPV)

KK: Ban Wa sub-district, Khon Kaen Province

SK: Tao Ngoi sub-district, Sakon Nakhon Province

CP: Nong Khon Thai sub-district, Chaiyaphum Province

When using combined methods as a composite standard, the performance of FECT in terms of sensitivities was 54%-85%, with the overall sensitivity of 63.6%. The specificities were 60%-98% between localities, with the overall specificity of 94.8%. Urine antigen detection showed the overall sensitivity 86.2% and was 83%-86% between localities. In the case of copro-antigen detection, the overall sensitivity was 85.3% and 85%-89% between localities (Table 4).

**Table 4:** Diagnostic performance of antigen detection methods for urine and copro-antigen compared with the composite gold standard which combined FECT and antigen detection method.

Tested method	Locality	Sensitivity (95% CI)	Specificity (95% CI)	°PPV	°NPV
FECT	KK	54.1 (48.2–60.0)	98.9 (96.8–99.8)	98.1	66.6
	SK	58.4 (50.7–65.8)	96.3 (90.8–99.0)	96.2	59.1
	CP	85.2 (78.9–90.2)	60.0 (42.1–76.1)	91.1	45.7
	Overall	63.6 (59.7–67.4)	94.8 (92.3–96.8)	95.0	62.9
Urine antigen detection	KK	86.8 (82.2–90.6)	93.0 (89.4–95.7)	92.2	88.0
	SK	84.8 (78.3–89.9)	88.9 (81.7–93.9)	91.4	80.6
	CP	83.1 (76.8–88.2)	81.0 (58.1–94.6)	97.4	35.4
	Overall	86.2 (83.2–88.8)	91.3 (88.3–93.8)	93.5	80.8
Copro-antigen detection	KK	89.4 (85.2–92.8)	93.3 (89.7–95.9)	92.8	90.1
	SK	87.0 (80.9–91.8)	87.4 (80.1–92.8)	90.4	83.2
	CP	85.3 (79.3–90.0)	81.0 (88.1–94.6)	97.5	38.6
	Overall	88.1 (85.3–90.6)	90.9 (87.8–93.5)	93.4	83.4

°Positive Predictive Value (PPV), Negative Predictive Value (NPV)

KK: Ban Wa sub-district, Khon Kaen Province

SK: Tao Ngoi sub-district, Sakon Nakhon Province

CP: Nong Khon Thai sub-district, Chaiyaphum Province

**Fig 5:**
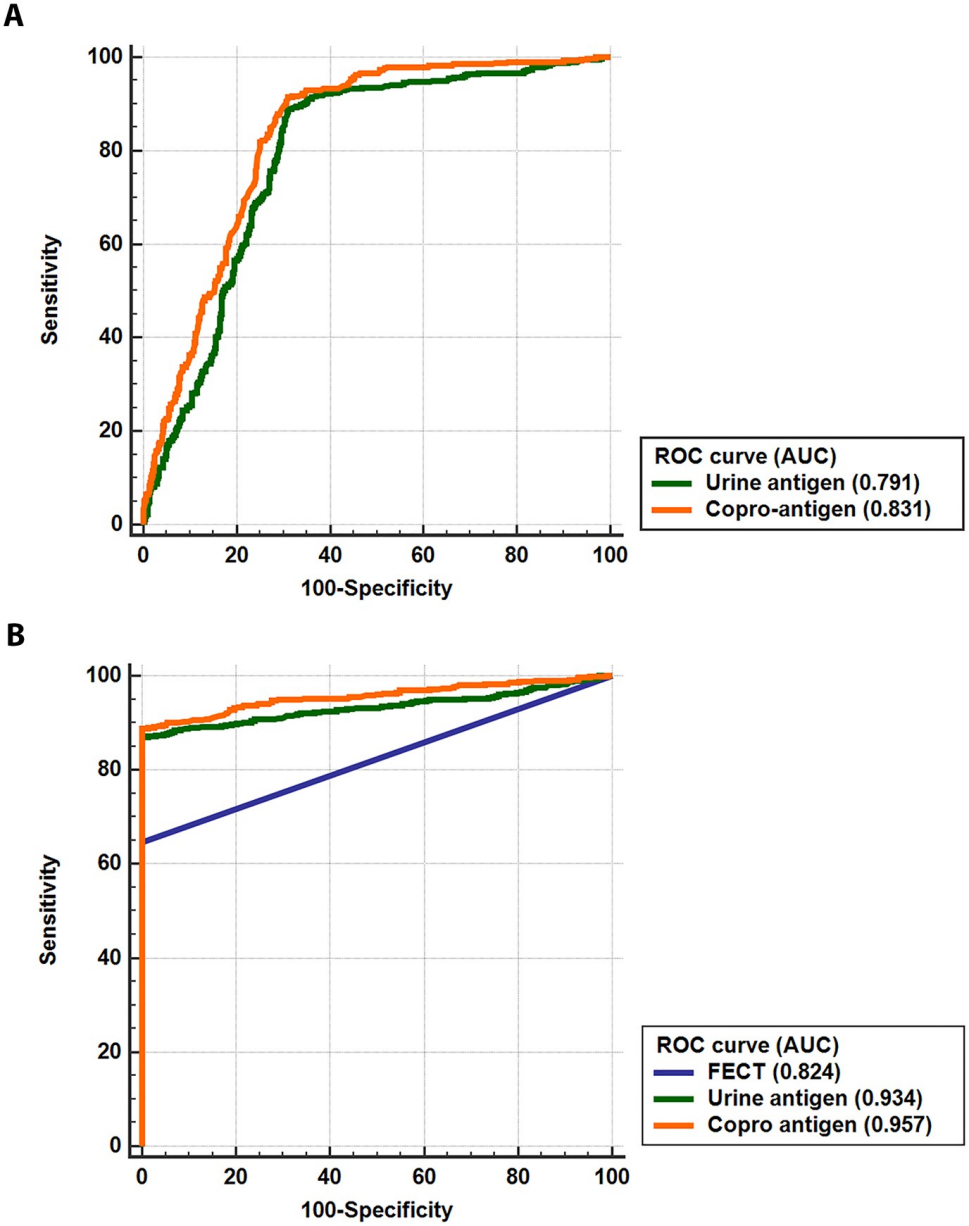
Receiver operating characteristic (ROC) curves comparing the antigen detection methods to the gold standard FECT (n = 1,043). (A) The ROC curve demonstrates the diagnostic performance of urine and copro-antigen detections with reference to the gold standard FECT. The AUCs of the urine and copro-antigen detection methods were 0.791 and 0.831, respectively. (B) The ROC curve shows the diagnostic performance of urine, copro-antigen detection and FECT, compared to the composite gold standard FECT which combined FECT and an antigen detection method. The AUCs for FECT, urine and copro-antigen detection and were of 0.824, 0.934 and 0.957, respectively.

For ROC curve analysis, with reference to FECT, the AUC for urine antigen detection was 0.791 while it was 0.831 for copro-antigen detection (Fig 5A). Based on the composite gold standard, the AUCs for the diagnostic assay were 0.824, 0.934 and 0.957 for FECT, urine and copro-antigen, respectively (Fig 5B).

The test for agreement between FECT versus urine antigen detection and FECT versus copro-antigen detection revealed moderate agreement, Kappa values (κ) were 0.547 and 0.570, respectively. The kappa tests showed good agreement (0.770) between urine and copro-antigen detection (Table 5).

**Table 5:** Agreement (Kappa value) of diagnosis between the FECT and antigen detection methods.

Variable	Kappa	95% Confidence Interval		P-value
		Lower	Upper	
FECT vs urine antigen detection	0.547	0.496	0.598	<0.001
FECT vs copro-antigen detection	0.570	0.522	0.616	<0.001
Urine vs copro-antigen detection	0.770	0.730	0.808	<0.001

Additionally, a logistic regression analysis was performed to predict the risk of human opisthorchiasis (odds ratios) based on increasing arbitrary unit of the antigen in urine and fecal samples by mAb-ELISA. The arbitrary unit was defined as the antigen concentration of 28.050 ng/ml for urine antigen and 75.729 ng/ml that predict the odd of one in having opisthorchiasis. The analysis showed that the odds ratio values increased according to the increasing concentration of both urine and copro-antigens (Table 6).

**Table 6:** Prediction of human opisthorchiasis by increasing urine and copro-antigen concentration.

**Unit increasing in urine antigen**	**Odds ratio**	**95% Confidence Interval**	
		**Lower**	**Upper**
1	1.042	0.199	5.510
10	2.170	1.377	3.421
20	2.393	1.029	5.568
30	9.454	4.245	21.056
**Unit increasing in copro- antigen**	**Odds ratio**	**95% Confidence Interval**	
		**Lower**	**Upper**
1	1.068	0.175	6.524
10	4.565	2.559	8.144
20	5.162	2.663	10.007
30	14.003	5.901	32.227

### Cross reactivity of urine and copro-antigen detection methods

To determine the specificity of mAb-ELISA, we applied this method to a separate set of urine and fecal extracts derived from participants with known parasite infection other than *O*. *viverrini* by the gold standard FECT.

**Fig 6:**
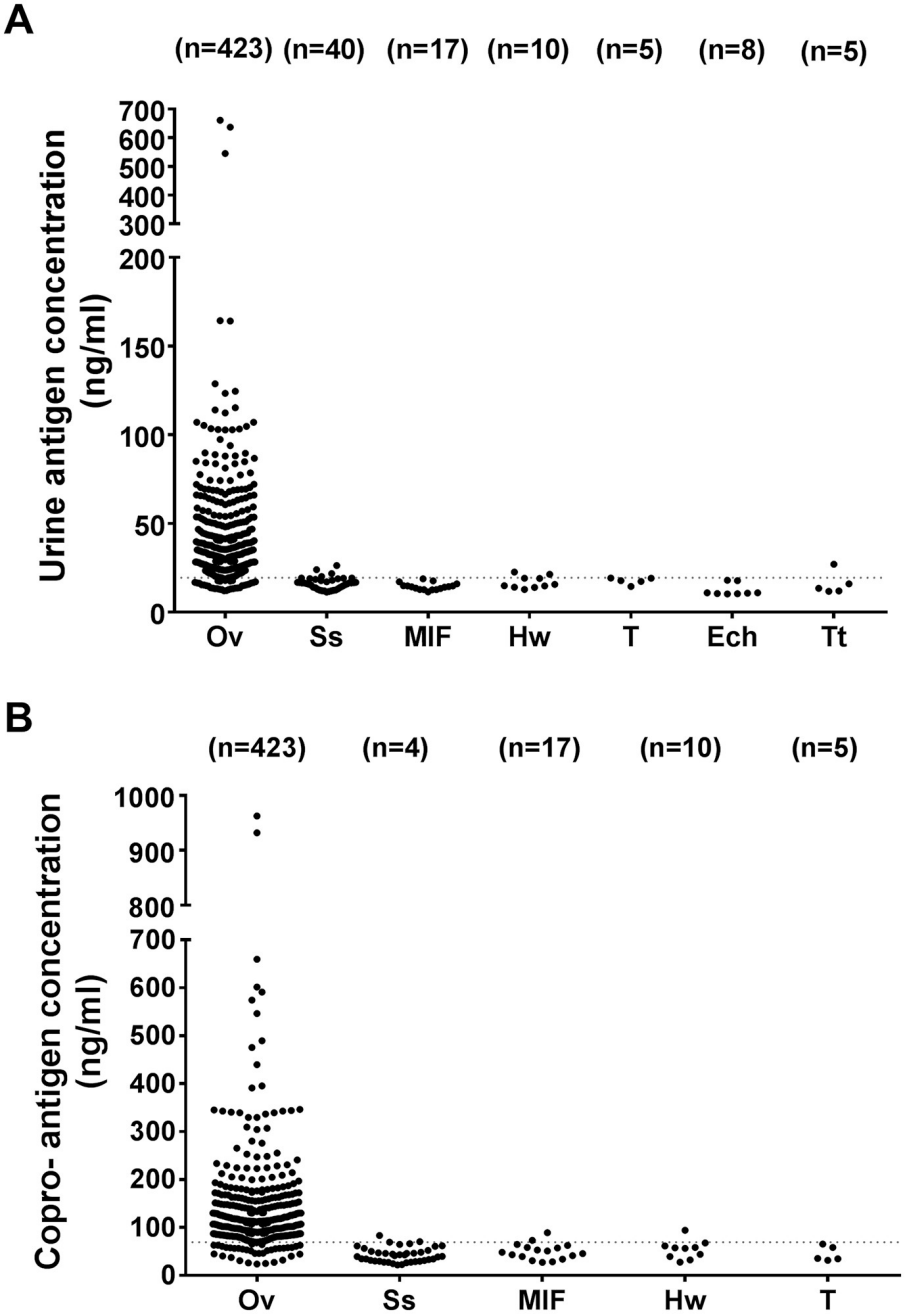
Tests for cross reactivity with other helminth infections by antigen assay using mAb-ELISA for opisthorchiasis (A) urine antigen detection and (B) copro-antigen detection method. Data shown are observed values of antigen/mL and the dotted-lined represents the cutoff values. Ov, *O*. *viverrini*; Ss, *S*. *stercoralis*; MIF, minute intestinal flukes; Hw, hookworm, T, *Taenia* sp.; Ech, *Echinostoma* sp. and Tt; *Tricuris trichiura*.

For the urine antigen detection method, a positive result was found in 10% of subjects with *Strongyloides stercoralis* infection, 20% (2/10) in subjects with hookworm infection and 20% (1/5) in subjects with *Trichuris trichiura* infection. (Fig 6A). Copro-antigen detection showed a positive result in 7.5% (3/40) in subjects with *Strongyloides stercoralis* infection, 11.7% (2/17) in minute intestinal flukes infection and 10% (1/10) in hookworm infection (Fig 6B).

## Discussion

In order to overcome the drawbacks of conventional fecal examination, the urine assay for *O*. *viverrini* antigen detection offers not only higher diagnostic accuracy, but also the practical advantage of utilizing urine instead of feces for the diagnosis of opisthorchiasis [28]. The urine antigen detection method is based on the hypothesis that excretory-secretory antigens released by living parasites can enter the blood circulation and be excreted via the kidneys into the urine. Herein, we applied this method in a community-based epidemiological study of opisthorchiasis, together with copro-antigen detection, using a mAb-ELISA protocol. The results indicate that urine as well as copro-antigen detection have comparable diagnostic accuracy and both methods have a superior diagnostic performance to that of fecal examination by FECT. The estimated concentration of antigen in the urine as well as that in feces significantly positively correlated with the intensity of infection measured by fecal egg count (egg/gram feces). Moreover, the antigen concentration in urine positively correlated with the levels antigen in the feces and the agreement test between methods showed good concordance (kappa value; 0.770). The average antigen concentrations in both urine and feces paralleled EPG. The antigen concentration in urine was two- to three- folds less than those in feces. On average of the antigen concentration in feces was 2.6 times that of urine. Because of the qualitative and quantitative concordance between urine and copro-antigen detection, as well as that from FECT, the ease of sample collection and specimen handling strongly indicate that urine detection is desirable for the diagnosis and screening of opisthorchiasis.

There was a higher positive detection obtained by urine (46–77%) and copro-antigen detection (48–78%) compared to FECT (29–77%). The discrepancies of antigen detection versus fecal examination by FECT were seen at low to, moderate intensities of opisthorchiasis, i.e. in SK a geometric mean of 25.3 EPG, in KK a geometric mean of 59.7 EPG. By contrast, the positive rates detected by both urine (77%) and copro-antigen detections (78%) were similar to FECT (77%) in the locality with high transmission intensity (geometric mean 127.1 EPG) in CP. These findings further support the usefulness of the urine antigen detection assay for screening for opisthorchiasis with low intensities of infection. This may help in avoiding the under diagnosis of the majority of individuals who have a low worm burden. However, whether our antigen detection assays can demonstrate a single worm infection, as reported in schistosomiasis [33], is not known, but this is a critical threshold to assess the impact of parasite control and eventually elimination, and hence requires further investigation. Moreover, the occurrence of varying transmission levels observed in the three study communities reflects once again the changing landscape of infection associated with multiple factors such as mass drug administration (MDA) and public health educational efforts [2, 34]. Therefore, a detection method which is analytically sensitive yet also rapid and easy to apply is required to monitor the current endemic situation of opisthorchiasis [7].

For cases of *O*. *viverrini* egg negativity as determined by FECT (n = 620), 191 (30.8%) and 195 (31.4%) positive cases were discovered by urine and copro-antigen detection, respectively. The finding of antigen positivity in this scenario indicates the vital role of worms rather than eggs as a source of antigen in the urine and feces in opisthorchiasis. A similar situation was previously observed in an autopsy study which revealed that fecal eggs were not discovered in the majority of infected individuals with less than 20 worms [35]. Further study is required to monitor the changes in antigen profile after treatment to justify the need for chemotherapy of the antigen positive but egg-negative individuals.

As opposed to egg negative cases, in cases of positive for *O*. *viverrini* eggs (1–100 EPG), not all individuals were found positive by the antigen detection methods. Within the egg positive group (n = 423), 11.3% were negative in urine and slightly less (8.5%) were copro-antigen negative. The explanation for the under diagnosis by antigen detections is currently not known but may depend on several possibilities. First, as the antigens appearing in the urine and feces are excretory-secretory products originating from living adult worms and may not directly relate to fecal egg count. Second, the presence of urine antigen may rely on the intermittent production of secretory products from the adult worms and also the passage through kidney glomerular filtration as proteinuria [36] and motility of gastrointestinal system in the case of copro-antigen. Thus, liver fluke-induced pathology in the biliary system and abnormal glomerular filtration in the kidneys may contribute in fluctuations of urine antigen. Third, the physical properties of urine and feces such as water content and fecal mass may influence the antigen concentration and eventually detectable antigen levels. Dilution effects of urine were addressed using the concentration procedure to find the accurate antigen detection levels in urine in schistosomiasis [33]. Lastly, more antigen positive cases can be found by multiple or repeated examination of the urine and feces over consecutive days, which yield a higher positive diagnostic rate as shown in the case of schistosomiasis [37, 38]. Kidney involvement in opisthorchiasis, in terms of proteinuria and deterioration of renal function associated with immune complex, has been shown previously [39, 40]. Since the antigen detection approach in opisthorchiasis is at a rudimentary phase, further work to prove and disprove the above hypotheses is required to augment our understanding of the presence as well as the pathway of urinary antigens, and hence the improvement of diagnostic accuracy.

The urine antigen detections had a 0.79 AUC and 89% sensitivity while the copro-antigen detection had a comparable accuracy (0.83 AUC and 90% sensitivity) with respect to the conventional FECT gold standard. A similar trend but with slightly lower diagnostic values was observed when the composite diagnosis was used as a reference standard in which FECT has the lowest sensitivity (63%) but high specificity. The observed diagnostic values in the current study were comparable to our recent study for urine [28] and copro-antigen [27] but higher than that in previous reports for copro-antigen detections [24, 25]. This observation is probably due to different mAb-ELISA protocols, particularly with monoclonal antibody being used. Additionally, the diagnostic accuracy of opisthorchiasis may approach a new gold standard when antigen detection is combined with fecal examination, agreeing with an approach used in cases of clonorchiasis [41].

Although *O*. *viverrini*-specific mAb was used for antigen detection in ELISA, cross relativities with other parasite antigens in urine were observed in individuals infected with *S*. *stercoralis*, hookworms and *T*. *trichiura*. In the case of copro-antigen detection, cross reactions occurred with *S*. *stercoralis*, MIF and hookworms. These findings are similar to our previous reports [27, 28], and may be explained by the fact that these patients probably harbored a low intensity of *O*. *viverrini* infection which could not be detected by FECT. Since multiple parasite infections are common in the study areas in Thailand, further cross reaction studies on the participants from non-*O*. *viverrini* endemic areas are needed.

In addition to cross reactivity with other parasitic infections, there are drawbacks for both the urine and copro-antigen detection methods that required attention. The main drawback is that the whole process of mAb-ELISA takes approximately 5–6 hours to complete and these assays needed experienced operators and special equipment. Furthermore, samples should be kept cold (4°C or on ice) during transportation and require centrifugation and pre-treatment with TCA prior to the analyses. Therefore, a “rapid point of care and ease-of-use” platform such as a lateral flow immunochromatgraphic strip or dipstick, which can be operated at point of care setting, should be developed.

### Conclusion

In this study, we show that *O*. *viverrini* antigen detection in urine and fecal samples has a higher diagnostic accuracy than conventional fecal examination method, FECT. Both antigen detection methods yielded comparable diagnostic accuracy and were significantly positively correlated. With high diagnostic accuracy and the ease of sample collection, the urine antigen detection method is a powerful approach for the diagnosis and population screening of opisthorchiasis in a clinical and also field setting. Pending additional study, the urine antigen detection method may serve not only the purpose diagnosis and screening, but also as a tool to follow-up opisthorchiasis patients to determine effective drug treatment in the control and elimination program.

## Supporting information

S1 Fig Linear relationship between urine (S1 A) and copro (S1 B) spiked excretory-secretory antigen of *O*. *viverrini* and OD values. The range of antigen used was 0.1–5000 ng/ml and OD values were obtained from the mAb-ELISA. Data shown are observed OD values. The solid lines represent the best-fit linear regression equations (P <0.001).
(TIF)

S1 Checklist (DOCX)
