# Supplementary figures and images for: Comparing the performance of urine and copro-antigen detection in evaluating Opisthorchis viverrini infection in communities with different transmission levels in Northeast Thailand
Source: PLoS Negl Trop Dis. 2019 Feb 8;13(2):e0007186. doi: 10.1371/journal.pntd.0007186 (PMC6383950; doi:10.1371/journal.pntd.0007186)

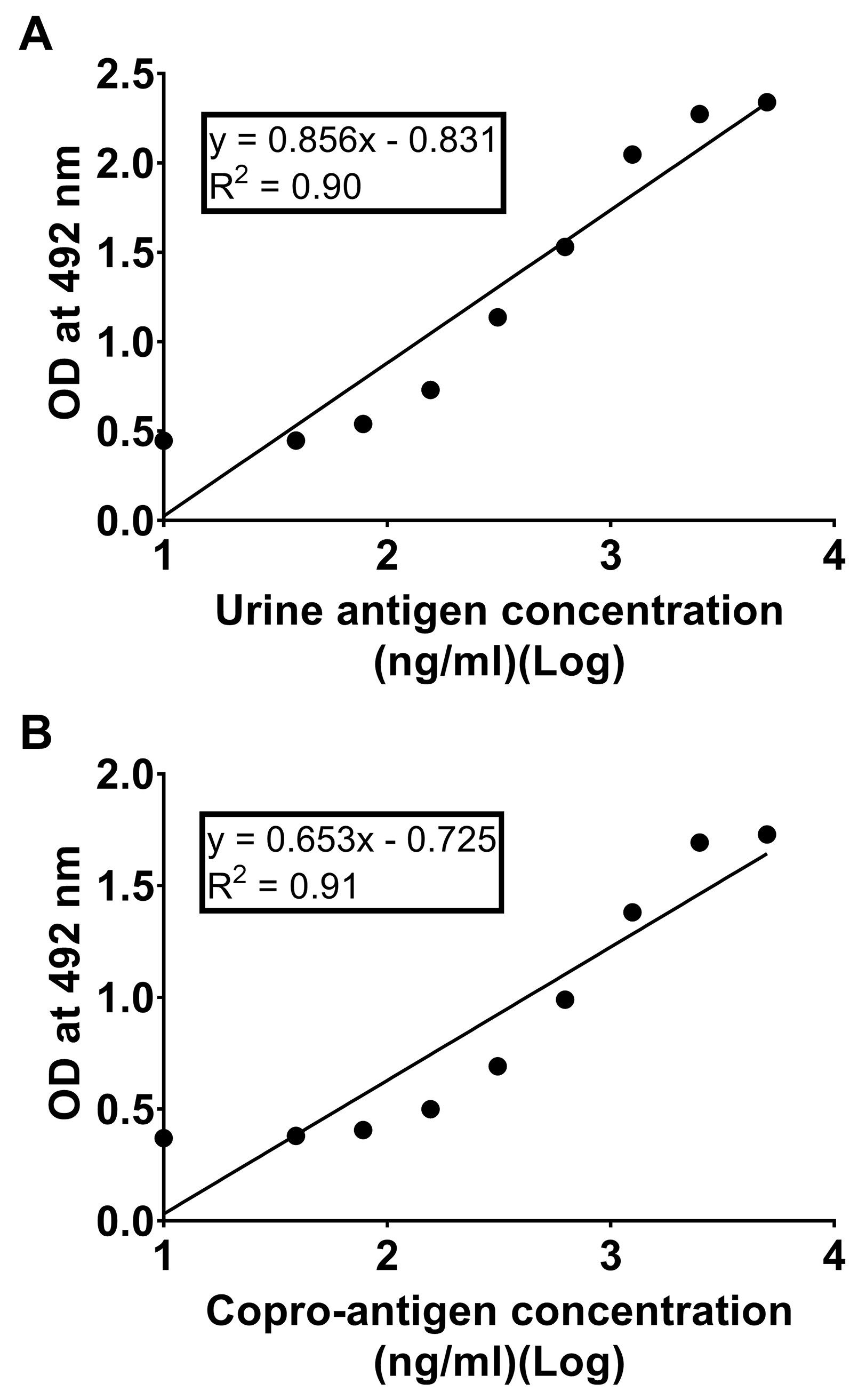

Supplement: S1 Fig — The range of antigen used was 0.1–5000 ng/ml and OD values were obtained from the mAb-ELISA. Data shown are observed OD values. The solid lines represent the best-fit linear regression equations (P <0.001). (TIF) [file pntd.0007186.s001.tif]
